# Metabolic Characterization of the Common Marmoset (*Callithrix jacchus*)

**DOI:** 10.1371/journal.pone.0142916

**Published:** 2015-11-18

**Authors:** Young-Mi Go, Yongliang Liang, Karan Uppal, Quinlyn A. Soltow, Daniel E. L. Promislow, Lynn M. Wachtman, Dean P. Jones

**Affiliations:** 1 Division of Pulmonary, Allergy and Critical Care Medicine, Department of Medicine, Clinical Biomarkers Laboratory, Emory University, Atlanta, Georgia, 30322, United States of America; 2 Department of Pathology and Department of Biology, University of Washington, Seattle, Washington, 98195, United States of America; 3 New England Primate Research Center, Harvard University, Southborough, Massachusetts, 01772, United States of America; National Eye Institute, UNITED STATES

## Abstract

High-resolution metabolomics has created opportunity to integrate nutrition and metabolism into genetic studies to improve understanding of the diverse radiation of primate species. At present, however, there is very little information to help guide experimental design for study of wild populations. In a previous non-targeted metabolomics study of common marmosets (*Callithrix jacchus*), *Rhesus macaques*, humans, and four non-primate mammalian species, we found that essential amino acids (AA) and other central metabolites had interspecies variation similar to intraspecies variation while non-essential AA, environmental chemicals and catabolic waste products had greater interspecies variation. The present study was designed to test whether 55 plasma metabolites, including both nutritionally essential and non-essential metabolites and catabolic products, differ in concentration in common marmosets and humans. Significant differences were present for more than half of the metabolites analyzed and included AA, vitamins and central lipid metabolites, as well as for catabolic products of AA, nucleotides, energy metabolism and heme. Three environmental chemicals were present at low nanomolar concentrations but did not differ between species. Sex and age differences in marmosets were present for AA and nucleotide metabolism and warrant additional study. Overall, the results suggest that quantitative, targeted metabolomics can provide a useful complement to non-targeted metabolomics for studies of diet and environment interactions in primate evolution.

## Introduction

Studies of primate genetics, nutrition and social behaviors have considerably advanced understanding of primate evolution [1–5], yet understanding of impact of diet and environmental factors affecting metabolism, such as water and food availability, infections, and predation, is incomplete. In recent years, metabolomics methods have become available, but metabolic studies of wild populations are challenging because of variable and non-uniform diets as well as unknown effects of age, sex, infections and threats of predation. Studies of captive primate colonies maintained for the study of health, aging and chronic disease provide an approach to establish baseline information with relatively controlled diet and environmental threats.

Common marmoset (*Callithrix jacchus*) is a New World monkey that belongs to the monophyletic primate family Callitrichidae with characteristics of small size, early maturation and relatively short lifespan [6, 7]. Among the Callitrichid family including marmosets, tamarins, lion tamarins and Goeldi’s monkey, marmosets are more likely to feed extensively on gums. Marmosets have dental adaptations to gouge trees and stimulate the flow of gum [8, 9], which allowed marmosets to colonize drier forests and small forest fragments where there is little fruit [10]. Laboratory marmosets are not a perfect model for wild animals because they tend to maintain higher body weights (350–400 g) compared to marmosets in the wild (320–340 g) [6] and are not exposed to their natural diet or habitat. Diet-dependent early-onset weight gain and obesity also occur with laboratory diets fed to marmosets [11] and progress is ongoing to improve captive management through development of evidence-based, standardized procedures for diet and feeding husbandry [11–16]. An earlier study by Tardif *et al*. also showed that infant-care factors such as being off carrier and weaning significantly affected growth rate of common marmosets suggesting that diet and social system are critical factor for metabolic control [17].

In a previous study, we used non-targeted metabolomics to compare metabolism of common marmoset (*Callithrix jacchus)* to six other mammalian species including *Rhesus macaque*, humans, and four non-primate mammalian species [18]. This non-targeted analysis using liquid chromatography and high-resolution mass spectrometry enables measurement of metabolites in 146 out of 154 metabolic pathways in the Kyoto Encyclopedia of Genes and Genomes (KEGG) database in a single analysis [19, 20]. This information-rich metabolic data can be combined with genomic or proteomic changes to provide an integrated perspective of biological phenotypes and improve understanding of the natural biology of organisms as well as the metabolic basis of disease. Tools are becoming available to address the complexities of omics data and the demanding biostatistical and bioinformatics for their interpretation [21]. Accumulating data with clinical and animal studies show metabolic alterations associated with multiple diseases of potential interest concerning primate biology, such as infectious diseases [22–24], diabetes [25], eye diseases [26], cardiovascular disease [27] and environmental toxicities [28].

In the comparative study of mammalian species, we found that even though humans have relatively uncontrolled environments compared to animals in research facilities, a similar number and diversity of chemicals was found in plasma. In an effort to identify plasma metabolites that could be useful for comparative biochemistry and toxicology research, we applied probability-based modularity clustering to identify metabolites with similar inter-species and intraspecies variation. Results showed that metabolite patterns were most similar within species and separated primates from other mammalian orders and families. Importantly, these characteristics suggest that quantitative differences in metabolite concentrations between species could provide useful information about nutrition and metabolism as determinants of primate evolution.

In the present study, we used a targeted metabolomics approach to test whether 55 plasma metabolites, including both nutritionally essential AA and non-essential metabolites and catabolic products, differ in concentration between common marmosets and humans. These were selected to include common clinical measures of disease (glucose, creatinine, urea, bilirubin), water-soluble vitamins (riboflavin, thiamin, niacin, niacinamide, pyridoxine, pyridoxamine, pyridoxal, pantothenic acid and biotin), amino acids and related metabolites, lipid-related metabolites (choline, betaine, dimethylglycine, carnitine, acetylcarnitine, sphingosine, sphinganine), nucleotide metabolites (hypoxanthine uric acid, allantoin) and environmental chemicals (flame retardant, triethylphosphate; an insecticide, pirimicarb; a plasticizer, dibutylphthalate). A multivariate analysis of covariance (MANCOVA) was further used to determine whether abundance of metabolites was affected by age, sex, body mass. The analytic methods with liquid chromatography and high-resolution mass spectrometry are similar to non-targeted metabolomics but have an advantage in supporting absolute quantification rather than relative quantification [19, 20]. In principle, the quantitative methods tested here can be extended to measure metabolites in 146 out of 154 metabolic pathways in the Kyoto Encyclopedia of Genes and Genomes (KEGG) database [21] and thereby provide functional measures to complement genomic studies. For the study, plasma from 50 captive marmosets was collected during routine health examination, and plasma metabolomics results were compared to plasma values for 80 humans [29].

## Materials and Methods

### Ethics Statement

This study complied with protocol approved by Harvard Medical School’s Standing Committee and Harvard Medical School’s Internal Animal Care and Use Committee for this specific study, and was not a general set of protocols. All procedures followed the American Society of Primatologists’ Principles for the Ethical Treatment of Nonhuman Primates. No animals were sacrificed as a result of the present study. This study contains original research on plasma metabolomics of common marmosets.

### Animals and Housing

Common marmosets (Callithrix jacchus) ranging in age from 2 to 15 years were maintained at the New England Primate Research Center (Southborough, MA, USA), an AAALAC-accredited facility, in accordance with the Guide for Care and Use of Laboratory Animals (“The Guide”) [30]. Colony animals were maintained under an animal holding and breeding protocol approved by Harvard Medical School’s Standing Committee on Animals; husbandry of the colony was described previously [31, 32]. The animals were fed commercial marmoset food (New World Primate Chow 8791, Harlan Teklad, Indianapolis, IN, USA), supplemented daily with a combination of fresh fruits, vegetables, seeds, eggs and/or mealworms. The animals had water ad libitum in polycarbonate water bottles. Animals were pair housed [standard cage, 5.8 ft^2^ (0.54 m^2^) of floor space, 2.7 ft (0.82 m) of height and equates to 15.7 ft^3^ (0.45 m^3^) of volume] or group housed in family units [aviary style cage, maximum of 9 individuals, 11.9 ft^2^ (1.10 m^2^) of floor space, 5.2 ft (1.58 m) of height and equates to 61.9 ft^3^ (1.75 m^3^) of volume]. In very rare instances, colony animals may have been temporarily housed singly for clinical reasons or while a suitable cage mate was identified. Caging, comprised of stainless steel mesh, met or exceeded the cage size requirements proposed by “The Guide” when “overall cage volume and linear perch space” were considered [30]. In addition to social housing, environmental enrichment consisted of cage fixtures for perching and hiding, nesting boxes, manipulanda, foraging opportunities, classical music and other veterinary approved auditory stimuli, positive human interaction, and others. Blood samples were collected during a quarterly physical examination; animals were sedated with 0.2 mL of ketamine given intramuscularly. Blood was collected in EDTA-containing evacuated tubes; plasma was separated, frozen, shipped on dry ice and maintained at −80°C until analysis.

### Metabolite Analysis by Liquid Chromatography-Mass Spectrometry (LC-MS)

Metabolites were analyzed by LC-MS using external calibration with authentic standards [20]. Briefly, 50 μL of plasma was added to 100 μL of acetonitrile and 2.5 μL of a mixture of 14 stable isotope standards. Samples were mixed, incubated at 4°C for 30 min, and centrifuged to remove proteins. Supernatants of samples were analyzed in triplicate by LC-MS [20, 33]. Data were extracted using apLCMS [16] with modifications by xMSanalyzer [34] as *m/z* features, where an *m/z* feature is defined by *m/z* (mass-to-charge ratio), rt (retention time) and ion intensity, and provided in the Supporting Information file (S1 Table). Metabolite identities were confirmed via rt relative to authentic chemicals and ion dissociation mass spectrometry (MS/MS) with fragmentation patterns matching those of authentic chemicals or those available from online databases, e.g., Scripps Center for Metabolomics (https://metlin.scripps.edu/index.php) and Human Metabolome Database (http://www.hmdb.ca/). Concentrations are expressed as mean ± standard deviation (SD) except as labeled otherwise.

### Human Metabolite Concentration Data

The data of human metabolites were obtained using the same methods as described for marmosets. Data for the present study were derived from EDTA plasma samples from 80 healthy humans analyzed as part of an ongoing healthy aging study (A.A. Quyyumi, Emory IRB protocol # IRB00024767)[29]. The study included individuals between 30 and 90 y, with even distribution of sex, and included individuals of different races and ethnicities present in the Atlanta area. All individuals were extensively screened to assure good health in terms of the absence of disease markers and absence of therapeutic drug use. Because the samples were de-identified and analysis was randomized and blinded, no additional details are available. Plasma was stored at -80°C before LCMS analysis. Additional reference values for many of the metabolites are available from HMDB (http://www.hmdb.ca/metabolites).

### Metabolic Pathways Analysis Using Kyoto Encyclopedia of Genes and Genomes (KEGG)

Quantitatively different metabolites were visualized in KEGG metabolic pathway analysis (http://www.genome.jp/kegg/pathway.html#metabolism).

### Statistics

For ANOVA (StatPlus) comparisons according to age and sex, the assumption of equal variances was evaluated using Levene's test of homogeneity. For each metabolite except Phe, the equal variance assumption was satisfied. For Phe, the equal variance assumption was met after Log2 transformation. Following two-way ANOVA, comparisons among groups within each factor were compared using Scheffe contrast among pairs of means with p<0.05. Figures were generated using OriginLab (OriginLab Corporation, Northampton, MA, USA). To test the effect of age, sex, mass and their interaction on the abundance levels of metabolites, MANCOVA based on the Pillai-Bartlett statistic was performed on 45 metabolites of 50 marmosets simultaneously. The MANCOVA analysis was performed using the manova function in R. This was followed by univariate ANCOVA tests to explore the effects of individual features.

## Results

### Age

Analyses were performed on 24 females and 26 males from 2 to 15 y. The marmosets were healthy with no difference in age (females, 7.8 ± 4.0 y; males, 8.0 ± 3.9 y; *p* = 0.8). The mean ages of younger (4.5 ± 1.8 y) and older (11.1 ± 2.5 y) females were comparable to those of younger (4.5 ± 1.8 y) and older (11.1 ± 2.3 y) males.

### Body Mass

The mean body mass of 50 marmosets was 400 ± 44 g (female, 404± 50 g; male, 396± 39 g) with range from 323 g to 535g [n = 40 (female = 18, male = 22) < 435 g; n = 10 (female = 6, male = 4) > 435g].

### Clinical Measures

Plasma glucose was 4.01 ± 1.44 mM, not significantly different from humans and similar to HMDB human values (Table 1). Cortisol (10.2 ± 4.1 μM) and cortisone (0.51 ± 0.19 μM) were significantly higher than human values, as previously shown [35]. Values for creatinine (83 ± 24 μM) and urea (4.69 ± 2.15 mM), indicators of renal function, were significantly lower than human values (Table 1) but within normal ranges for human values in HMDB. Creatine was not significantly different from human values. Bilirubin (1.8 ± 1.1 μM), a measure that is increased with liver dysfunction, was significantly lower than human values (5.7 ± 6.4 μM).

**Table 1:** Plasma metabolite concentrations in common marmosets.

Metabolite	*m/z*	rt	Marmosets Mean±SD	HumansMean±SD	Human metabolomics Database
**Health indicators**					
Glucose (mM)	203.0512	57	4.0 ± 1.4	4.0±0.9	3.9 to 6.1
Creatine (μM)	132.0759	52	38 ± 15	52±35	8.4 to 65
Creatinine (μM)	114.0905	393	83 ± 24	102±16*	56 to 109
Urea (mM)	121.0711	58	4.7 ± 2.1	3.2±1.6*	4 to 9
Cortisol (μM)	363.2143	160	10.2 ± 4.1	0.49±0.25*	0.028 to 0.66
Cortisone (μM)	361.1991	174	0.51 ± 0.19	0.010±0.004*	0.022 to 0.075
Bilirubin (μM)	585.2668	350	1.9 ± 1.1	5.7 ± 6.4*	8 to 15
**Vitamins and Coenzymes**					
Riboflavin (nM)	377.1468	215	6.0 ± 5.7	15.5 ± 0.02*	5.4 to 28
Thiamine (μM)	265.1164	69	0.64 ± 0.62	0.39 ± 0.3	0.09 to 0.28
Niacin; nicotinic acid (μM)	124.0398	45	17.5 ± 7.0	29.8 ± 29.0	43 to 55
Nicotinamide (μM)	123.0544	326	0.43 ± 0.18	0.27 ± 0.15*	0.43 to 0.45
Methylnicotinic acid (nM)	138.0540	64	16 ± 9	20.0 ± 19.9	N/A
Pyridoxine (nM)	170.0714	61	59 ± 43	9.7 ± 6.1*	7 to 60
Pyridoxal (nM)	168.0643	93	230 ± 110	N/A	200 to 300
Pyridoxamine (nM)	169.0950	101	71 ± 56	0.14 ± 0.09*	126 to 202
Pantothenic acid (μM)	220.1761	231	4.1 ± 1.8	7.57 ± 7.22*	4.5 to 5.3
Biotin (nM)	245.0980	289	2.7 ± 1.1	4.5 ± 3.3*	0.6 to 1.9
**Amino Acids**					
Arginine (μM)	175.1178	59	40 ± 29	72 ± 33*	60 to 140
Histidine (μM)	178.0575	51	71 ± 21	52 ±14*	75 to 143
Leucine/Isoleucine (μM)	176.0646	59	88 ± 43	156 ± 56*	155 to 355
Lysine (μM)	191.0755	48	104 ± 57	117 ± 41	178 to 434
Methionine (μM)	150.0568	79	39 ± 20	24 ± 6*	25 to 35
Phenylalanine (μM)	166.0851	57	271 ± 151	47 ± 15*	48 to 88
Threonine (μM)	120.0646	88	132 ± 39	103 ± 33*	102 to 260
Tryptophan (μM)	205.0958	56	72 ± 21	40 ± 11*	44 to 78
Asparagine (μM)	133.0679	92	18 ± 15	N/A	16 to 57
Citrulline (μM)	176.1018	63	34 ± 18	31 ± 14	27 to 38
Glutamate (μM)	148.0594	70	36 ± 25	23 ± 13	24 to 145
Glutamine (μM)	147.0758	56	553 ± 141	463 ± 317	490 to 645
Proline (μM)	116.0698	54	168 ± 62	195 ± 66	168 to 239
Taurine (μM)	148.0027	52	105 ± 37	32 ± 13*	42 to 162
Tyrosine (μM)	182.0799	58	44 ± 20	44 ± 11	54 to 143
**Amino Acid Metabolites**					
5-Hydroxytryptophan (μM)	221.0907	62	0.41 ± 0.32	0.030 ± 0.012*	0.015 to 0.021
Indoleacrylic acid (μM)	188.0693	64	0.02 ± 0.01	0.01 ± 0.00*	N/A
Indolelactate (μM)	206.0797	101	4.98 ± 1.67	0.72 ± 0.30*	0.5 to 5
3-Indolepropionic acid (μM)	190.0849	202	9.11 ± 5.33	0.04 ± 0.04*	0.29 to 1.09
Kynurenine (μM)	209.0914	73	3.68 ± 1.47	1.84 ± 0.72*	1.5 to 1.7
Phenylacetate (nM)	137.0586	216	2.7 ± 2.0	2.78 ± 2.15	N/A
Methylphenyllactate (nM)	181.0847	167	14 ± 7	48 ± 64*	N/A
Methylphenylpropanoate (nM)	165.0899	257	8.0 ± 5.0	6.5 ± 3.8	N/A
Homogentisic acid (μM)	169.0436	106	0.15 ± 0.09	0.02 ± 0.01*	0.014 to 0.071
Oxoproline (μM)	130.0490	53	47 ± 11	62 ± 13*	13 to 161
Hippurate (μM)	180.0644	63	8.6 ± 5.8	6.6 ± 6.3	0 to 5
2-Aminobutyrate (nM)	104.0698	54	6.0 ± 4.0	4.6 ± 1.4*	N/A
**Lipid-Related Metabolites**					
Choline (μM)	104.1062	51	1.5 ± 0.5	0.85 ± 0.23*	8.7 to 12.5
Betaine (μM)	118.0854	54	123 ± 64	31 ± 13*	20 to 144
Dimethylglycine (μM)	104.0697	424	2.91 ± 0.75	2.28 ± 0.97*	1.8 to 3.7
Carnitine (μM)	162.1114	79	6.7 ± 2.2	66 ± 24*	26 to 79
Acetylcarnitine (μM)	204.1216	66	0.54 ± 0.30	N/A	3.2 to 7.6
Sphinganine (nM)	302.3034	331	4.8 ± 2.6	6.6 ± 6.3	11
Sphingosine (μM)	300.2877	510	0.74 ± 0.33	1.21 ± 0.38*	0.05 to 0.51
**Nucleotide-Related Metabolites**					
Uridine (μM)	245.0757	95	2.11 ± 0.84	0.91 ± 0.68*	2.9 to 3.3
Hypoxanthine (μM)	137.0448	59	54 ± 34	3.9 ± 3.6*	1.3 to 54.5
Uric acid (μM)	169.0342	54	84 ± 73	214 ± 54*	238 to 506
Allantoin (μM)	159.0502	63	2.2 ± 1.1	N/A	1.0 to 3.2
**Environmental Chemicals**					
Triethylphosphate (nM)	183.0768	382	14.5 ± 3.4	10.8 ± 9.5	N/A
Pirimicarb (nM)	239.1475	548	0.7 ± 0.4	0.80 ± 0.36	N/A
Dibutylphthalate (nM)	279.1573	381	3.0 ± 1.0	14.0 ± 44.4	N/A

Plasma metabolites of common marmosets (n = 50) quantified by LC-MS are included along with measures for human plasma (n = 80) obtained with the same method. Ranges of concentrations of metabolites in human plasma reported by HMDB are also included for comparison. (*m/z*, mass-to-charge, rt, retention time). N/A, data not available.

* Levels in common marmosets significantly different from the levels in human, *p* < 0.05, following Bonferroni correction.

### Water-Soluble Vitamins

Plasma levels of water-soluble vitamins (riboflavin, thiamin, niacin, niacinamide, pyridoxine, pyridoxamine, pyridoxal, pantothenic acid and biotin) in marmosets overlapped with HMDB values for humans (Table 1), but riboflavin, nicotinamide, pyridoxine, pyridoxamine, pantothenate and biotin were significantly different between marmosets and humans (Table 1).

### Amino Acids

**Fig 1:**
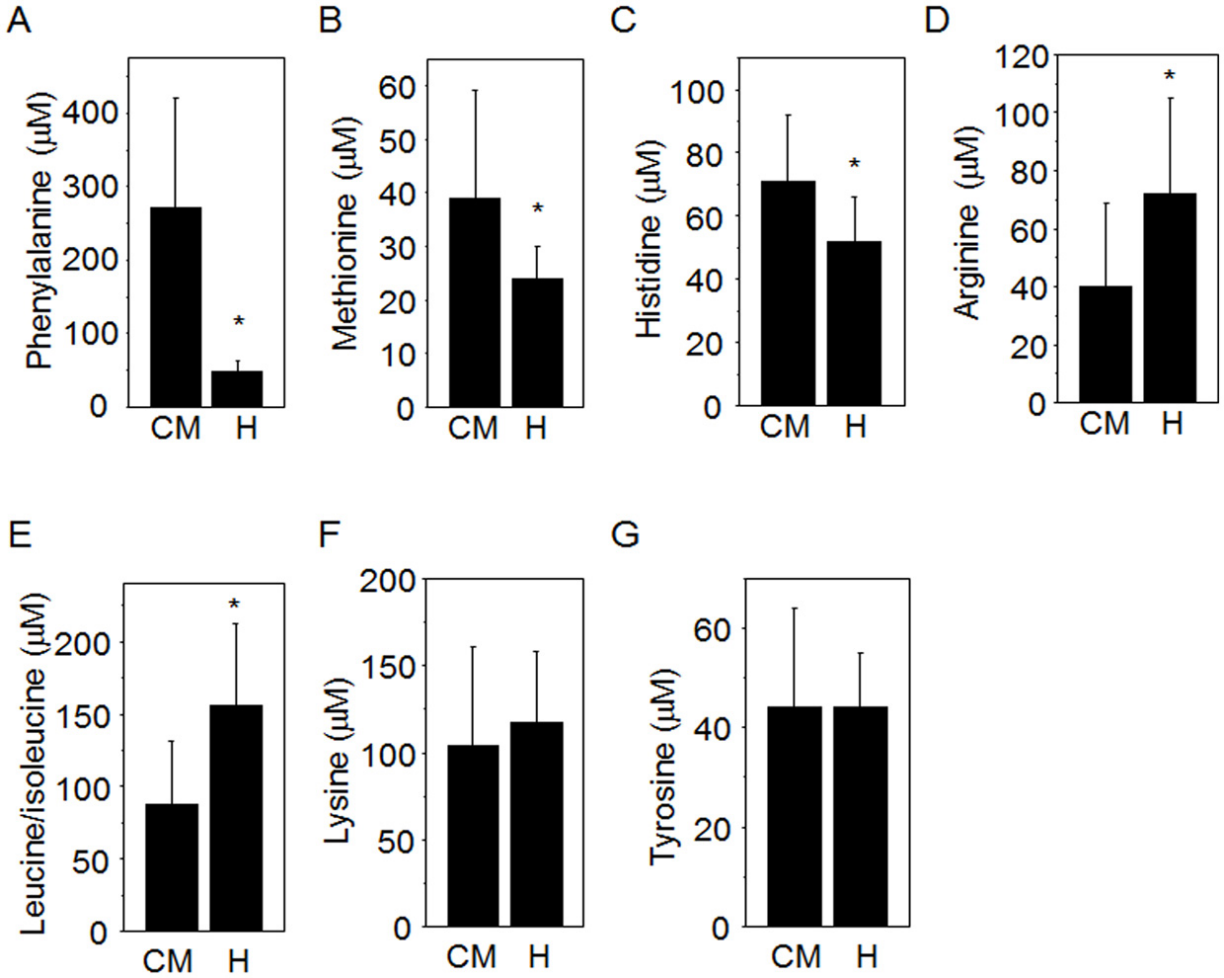
Plasma levels of amino acids in common marmosets and human. Plasma of common marmosets including female and male marmosets (n = 50) was analyzed for amino acids by LCMS to compare with values of human plasma (H). Concentration of phenylalanine (A) and methionine (B), histidine (C) of common marmoset (CM, mean ± SD) were significantly higher than human. Arginine (D) and leucine/isoleucine (E) were significantly lower in CM than H. Lysine (F) and tyrosine (G) values of CM were not significantly different from H (Mean ± SD). * *p* < 0.05.

As previously reported [33], the mean phenylalanine (Phe) concentration in plasma (271 ± 151 μM) was substantially higher than mean values for human plasma (47 ± 15 μM) and reference HMDB values (44 to 88 μM) (Fig 1A, Table 1). Mean plasma methionine (Met) was also significantly higher (Fig 1B, and respective mean values for arginine (Arg), histidine (His) and leucine/isoleucine (Leu/Ile), were significantly lower than human values (Fig 1C–1F, Table 1). Mean plasma threonine and tryptophan (Trp) values were within the range of mean values summarized in HMDB for humans but were significantly different in the direct comparison (Table 1). With the exception of taurine, which was higher in marmosets, all of the non-essential amino acids that were measured in marmoset plasma were not significantly different from human values and within the range of values summarized in HMDB for humans. Mean value for tyrosine (Fig 1G) was not significantly different from measured human values but was below the range of values in HMDB (Table 1).

### Amino Acid Metabolites

Five metabolites of Trp were measured, and all were significantly higher than values in humans (Table 1). Four Phe metabolites were quantified; phenylacetate and methylphenylpropionate were not different from human values, homogentisate (0.15 ± 0.09 μM) was higher than humans (0.02 ± 0.01 μM) and methyphenyllactate (14 ± 7 μM) was lower than humans (48 ± 64 μM). Mean plasma values for oxoproline (pyroglutamate) and aminobutyric acid significantly differed between marmosets and humans, but the ranges for both overlapped substantially. The glycine metabolite of benzoic acid, hippuric acid, was not significantly different between marmosets and humans.

### Choline and Related Metabolites

**Fig 2:**
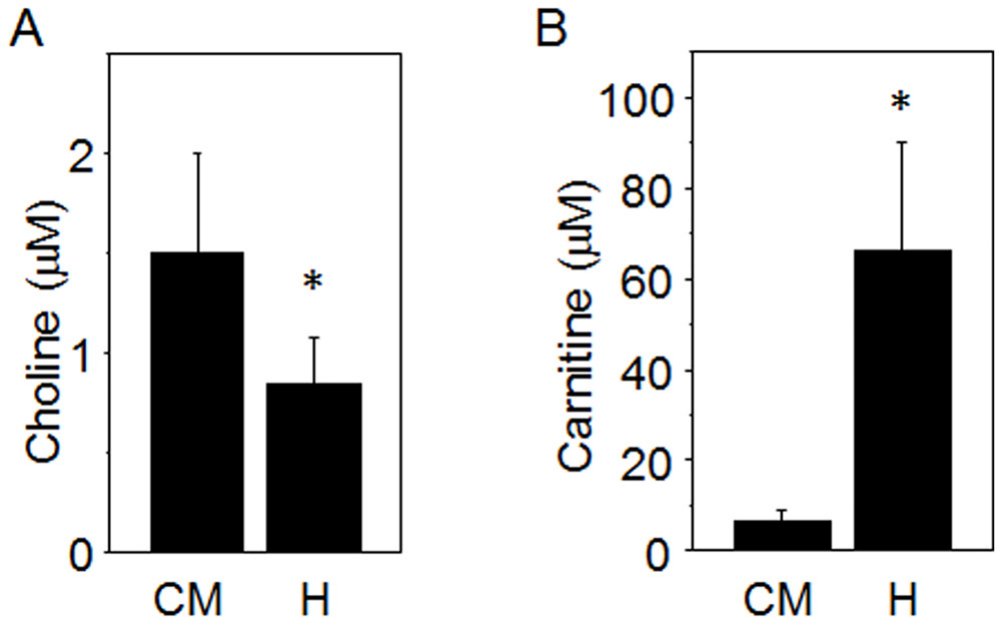
Choline and carnitine metabolites were lower in common marmoset than human. Using the same method described in Fig 1, choline (A) and carnitine (B) in lipid category of common marmoset (CM) were quantified and compared with human (H). Bar graphs show Mean ± SD. * *p* < 0.05.

Metabolites involved in 1-carbon metabolism, betaine and dimethylglycine, had values that were comparable to human values in HMDB, but mean values were significantly different when compared to measurements in humans (Table 1). In contrast, choline and carnitine metabolites were approximately 10-fold lower than human values (Fig 2, Table 1). These included data for marmosets and humans, respectively: choline 1.46 ± 0.51 μM, 8 to 13 μM; carnitine 6.7 ± 2.2 μM, 26 to 79 μM; and acetylcarnitine 0.54 ± 0.30 μM, 3.2 to 7.6 μM. Sphingosine (0.74 ± 0.33 μM) was significantly lower than humans (1.21 ± 0.38 μM) but the ranges overlapped. Sphinganine was not significantly different between marmosets and humans.

### Nucleotide Metabolites

Plasma uridine was higher than measured human values but similar to the HMDB range. Hypoxanthine was within the HMDB range but was 10-fold higher than the values in humans obtained with the same method. Uric acid concentration (84 ± 73 μM) was lower than values for humans (238 to 506 μM). Allantoin was not measured in humans but was within the range reported in HMDB (Table 1).

### Environmental Chemicals

A previous comparative study of mammalian species detected environmental agents in marmoset plasma, including a flame retardant, triethylphosphate, an insecticide, pirimicarb, and a plasticizer, dibutylphthalate. These chemicals were quantified and found to be present at 14.5 ± 3.4 nM, 0.7 ± 0.4 nM and 3.0 ± 1.0 nM, respectively, and were not significantly different from human values. Respective concentrations for human plasma were not available in HMDB or anywhere else. A literature value for dibutylphthalate concentration in human serum, 4.4 nM [36], was also similar to the value found in marmosets.

### Sex and Age-Dependent Differences in Marmoset Metabolites

Two-way ANOVA with post-hoc comparisons was performed for male and female marmosets divided according to age (2 to 7 y and 8 to 15 y). This division was based upon the age range of the colony and designed to give comparable numbers of older and younger animals. Data are provided in Table 2. To allow adjustment for body mass, MANCOVA and ANCOVA were also performed with age, sex and body mass. For these analyses, the R package limited the maximal number of metabolites that could be included to 45; thus, MANCOVA and ANCOVA analyses were performed without the lowest abundance groups of chemicals, *i*.*e*., vitamin metabolites and environmental chemicals. MANCOVA showed *p* values reflecting a trend for sex (*p* = 0.075) and age (*p* = 0.092), but not for body mass (p = 0.84). ANCOVA data are provided in Table 3. For simplicity, differences according to sex are discussed first followed by differences according to age. ANCOVA showed only three metabolites significantly associated with body mass after adjusting for sex and age; these were urea, kynurenine and homogentisate (Table 3).

**Table 2:** Marmoset plasma metabolite concentrations according to age group and sex.

Metabolite	Females<8 y	Females ≥8 y	Males <8y	Males ≥8y
**Clinical measures**				
Glucose (mM)	4.8 ± 1.8^a^	4.1 ± 1.1	3.5 ± 1.3^a^	3.8 ± 1.4
Creatine (μM)	31 ± 13	43 ± 12	38 ± 11	42 ± 20
Creatinine (μM)	74 ± 16	89 ± 34	88 ± 26	81 ± 18
Urea (mM)	3.7 ± 2.0^a^	3.7 ± 1.3^b^	5.5 ± 2.4^a^	5.7 ± 2.1^b^
Cortisol (μM)	11 ± 4	9 ± 5	11 ± 3	10 ± 4
Cortisone (μM)	0.59 ± 0.24	0.45 ± 0.16	0.57 ± 0.13^d^	0.44 ± 0.16^d^
Bilirubin (μM)	2.0 ± 1.1	2.1 ± 1.0	1.5 ± 0.8	1.9 ± 1.3
**Vitamins and Coenzymes**				
Riboflavin (nM)	5 ± 5	11 ± 10	4 ± 4	6 ± 4
Thiamine (μM)	0.61 ± 0.37	0.95 ± 0.81	0.37 ± 0.27	0.57 ± 0.68
Niacin; nicotinic acid (μM)	17 ± 6	19 ± 6	19 ± 10	15 ± 4
Nicotinamide (μM)	0.42 ± 0.23	0.46 ± 0.17	0.41 ± 0.16	0.42 ± 0.18
Methylnicotinic acid (nM)	20 ± 10	14 ± 6	17 ± 11	14 ± 6
Pyridoxine (nM)	33 ± 14	57 ± 39	63 ± 37	76 ± 57
Pyridoxal (nM)	0.19 ± 0.09	0.25 ± 0.12	0.28 ± 0.11	0.20 ± 0.10
Pyridoxamine (nM)	87 ± 83	61 ± 25	57 ± 39	75 ± 57
Pantothenic acid (μM)	4.2 ± 0.9	3.5 ± 2.1	4.0 ± 1.4	4.5 ± 2.4
Biotin (nM)	3.1 ± 1.4	2.5 ± 1.1	2.9 ± 1.2	2.2 ± 0.7
**Amino Acids**				
Arginine (μM)	35 ± 28	43 ± 28	32 ± 21	48 ± 36
Histidine (μM)	74 ± 27	81 ± 21	59 ± 14	72 ±19
Leucine/Isoleucine (μM)	99 ± 41	93 ± 58	87 ± 37	74 ± 33
Lysine (μM)	129 ± 82^a^	117 ± 48	76 ± 28^a^	96 ± 49
Methionine (μM)	47 ± 23^a^	49 ± 22^b^	29 ± 13^a^	32 ± 15^b^
Phenylalanine (μM)	348 ± 141^a^	404 ± 158^b^	167 ± 61^a^	180 ± 62^b^
Threonine (μM)	146 ± 58	135 ± 33	125 ± 31	124 ± 29
Tryptophan (μM)^e^	60 ± 20^a^	69 ± 19	90 ± 13^a^ ^,^ ^d^	71 ± 19^d^
Asparagine (μM)	16 ± 8	12 ± 11	23 ± 17	18 ± 18
Citrulline (μM)	35 ± 19	42 ± 17	32 ± 12	29 ± 20
Glutamate (μM)	216 ± 131	296 ± 180^b^	138 ± 83	138 ± 82^b^
Glutamine (μM)	509 ± 135	582 ± 168	519 ± 125	594 ± 130
Proline (μM)	169 ± 71	194 ± 58	158 ± 50	155 ± 67
Taurine (μM)	102 ± 43	107 ± 30	101 ± 35	110 ± 42
Tyrosine (μM)	45 ± 19	53 ± 20	41 ± 16	39 ± 22
**Amino Acid Metabolites**				
5-Hydroxytryptophan (μM)	0.35 ± 0.19	0.56 ± 0.52	0.31 ± 0.16	0.42 ± 0.27
Indoleacrylic acid (μM)^e^	13 ± 5^a^	15 ± 5	20 ± 4^a^ ^,^ ^d^	14 ± 4^d^
Indolelactate (μM)	4.9 ± 2.2	4.7 ± 1.0	5.3 ± 1.3	5.0 ± 2.0
3-Indolepropionic acid (μM)	9.5 ± 3.8	8.5 ± 6.8	9.8 ± 3.7	8.7 ± 6.6
Kynurenine (μM)	4.1 ± 1.9	3.4 ± 1.1	3.6 ± 1.4	3.6 ± 1.5
Phenylacetate (nM)	3.3 ± 1.9	2.8 ± 1.7	1.7 ± 1.5	2.8 ± 2.5
Methylphenyllactate (nM)	12 ± 5	15 ± 8	12 ± 6	17 ± 8
Methylphenylpropanoate (nM)	6.2 ± 3.8^a^	5.5 ± 2.4	10.9 ± 5.0^a^	9.0 ± 6.0
Homogentisic acid (μM)	139 ± 77	198 ± 119	113 ± 62	143 ± 100
Oxoproline (μM)	42 ± 8^c^	50 ± 13^c^	45 ± 7	50 ± 12
Hippurate (μM)	6.1 ± 2.6^c^	11.8 ± 8.7^c^	7.3 ± 4.2	9.2 ± 4.8
2-Aminobutyrate (nM)	5.3 ± 3.3	8.0 ± 5.4	6.7 ± 2.7	5.5 ± 2.4
**Lipid-Related Metabolites**				
Choline (μM)	1.4 ± 0.5	1.6 ± 0.5	1.3 ±0.5	1.5 ± 0.5
Betaine (μM)	139 ± 76	141 ± 79	116 ± 48	99 ± 45
Dimethylglycine (μM)	2.6 ± 0.9	2.9 ± 0.6	2.7 ± 0.7^d^	3.3 ± 0.7^d^
Carnitine (μM)	5.4 ± 1.4^c^	7.9 ± 2.6^c^	6.2 ± 1.9	7.0 ± 2.3
Acetylcarnitine (μM)	0.45 ± 0.15	0.57 ± 0.26	0.45 ± 0.22	0.65 ± 0.44
Sphinganine (nM)	6.0 ± 2.1^a^	5.0 ± 2.5	3.5 ± 2.6^a^	4.9 ± 2.7
Sphingosine (μM)	0.69 ± 0.34^a^	0.62 ± 0.33	1.00 ± 0.27^a^ ^,^ ^d^	0.69 ± 0.29^d^
**Nucleotide-Related Metabolites**				
Uridine (μM)	2.0 ± 0.7^c^	1.4 ± 0.6^b^ ^,^ ^c^	2.5 ± 0.9	2.4 ± 0.7^b^
Hypoxanthine (μM)	72 ± 40^c^	41 ± 20^c^	65 ± 40^d^	39 ± 21^d^
Uric acid (μM)	110 ± 120	60 ± 38	78 ± 41	78 ± 50
Allantoin (μM)^e^	2.7 ± 0.9^c^	1.5 ± 0.8^b^ ^,^ ^c^	2.0 ± 1.1	2.4 ± 1.4^b^
**Environmental Chemicals**				
Triethylphosphate (nM)	13.7 ± 3.7	14.8 ± 4.3	14.3 ± 2.7	15.0 ± 3.3
Pirimicarb (nM)	0.86 ± 0.49	0.63 ± 0.32	0.54 ± 0.33	0.73 ± 0.35
Dibutylphthalate (nM)	0.26 ± 0.14	0.25 ± 0.10	0.30 ± 0.13	0.33 ± 0.18

^a^Significant between younger females and younger males

^b^Significant between older females and older males

^c^Significant between younger females and older females

^d^Significant between younger males and older males

^e^Significant sex by age interaction

**Table 3:** ANCOVA *p*-values on feature-by-feature basis for association with age, sex, and body mass.

Metabolites	Sex	Age	Mass	Sex:Age	Sex:Mass	Age:Mass	Sex:Age:
Glucose	0.6702	0.6762	0.2750	0.8249	0.2300	0.4465	0.4823
Creatine	0.5185	0.1417	0.1169	0.5240	0.6274	0.2144	0.5511
Creatinine	0.5040	0.5988	0.2240	0.2689	0.8801	0.0149	0.1387
Urea	0.0275	0.6168	0.0329	0.6850	0.2055	0.5565	0.2932
Cortisol	0.3917	0.0146	0.8398	0.9025	0.8167	0.4715	0.9400
Cortisone	0.7795	0.0036	0.2087	0.4746	0.7381	0.8159	0.8223
Bilirubin	0.3447	0.3378	0.3038	0.4172	0.2819	0.6744	0.4230
Arginine	0.8798	0.9302	0.2729	0.8844	0.8192	0.3855	0.7448
Histidine	0.1126	0.1081	0.5190	0.4284	0.0445	0.2957	0.3725
Leu/Ile	0.8572	0.5710	0.3407	0.3482	0.2384	0.3233	0.7540
Lysine	0.3137	0.4783	0.6965	0.1785	0.4159	0.9617	0.9369
Methionine	0.0032	0.3223	0.7058	0.4598	0.9862	0.9116	0.8842
Phenylalanine	0.0000	0.5352	0.6947	0.9600	0.1480	0.8879	0.7786
Threonine	0.2645	0.6217	0.9771	0.8287	0.5194	0.1060	0.1923
Tryptophan	0.0027	0.0477	0.1598	0.0135	0.0275	0.1748	0.2667
Asparagine	0.1116	0.3840	0.3398	0.8173	0.1481	0.2459	0.5809
Citrulline	0.1063	0.8311	0.4934	0.1053	0.7440	0.2792	0.2082
Glutamate	0.0018	0.1411	0.1187	0.2060	0.0320	0.7792	0.9008
Glutamine	0.6311	0.0465	0.5603	0.6394	0.8829	0.9432	0.2904
Proline	0.2502	0.8600	0.3103	0.4235	0.6924	0.7948	0.4435
Taurine	0.8139	0.8464	0.1289	0.6052	0.5907	0.4969	0.3662
Tyrosine	0.0847	0.3915	0.4905	0.1991	0.4870	0.0983	0.8035
5-Hydroxy-tryptophan	0.3844	0.0414	0.1108	0.7027	0.6525	0.4743	0.6012
Indoleacrylic acid	0.0277	0.0047	0.1709	0.0044	0.1104	0.0722	0.2440
Indolelactate	0.2353	0.5699	0.4865	0.6913	0.6157	0.0912	0.2744
3-Indole-propionic acid	0.8339	0.0228	0.4181	0.2743	0.1895	0.4770	0.6788
Kynurenine	0.6791	0.5554	0.0159	0.4220	0.1512	0.9392	0.6270
Phenylacetate	0.2585	0.1280	0.6268	0.1764	0.5340	0.4608	0.0448
Methylphenyl-lactate	0.7493	0.5574	0.8937	0.6992	0.9230	0.4890	0.5715
Methylphenyl-propanoate	0.0018	0.5249	0.7533	0.6030	0.4451	0.4576	0.5756
Homogentisic acid	0.2096	0.0272	0.0411	0.7161	0.6945	0.7880	0.9114
Oxoproline	0.4567	0.0694	0.3533	0.9628	0.8214	0.5262	0.3992
Hippurate	0.9654	0.0172	0.1716	0.9664	0.5540	0.9190	0.7350
2-Aminobutyrate	0.6061	0.7001	0.7046	0.2406	0.1440	0.7918	0.4595
Choline	0.4389	0.3375	0.2244	0.4195	0.7784	0.5934	0.7220
Betaine	0.1900	0.3131	0.2656	0.5522	0.0149	0.3690	0.0665
n,n-Dimethylglycine	0.1114	0.0161	0.1260	0.8903	0.1852	0.6146	0.7978
Carnitine	0.4672	0.0058	0.2663	0.3918	0.1193	0.8618	0.4059
Acetyl-carnitine	0.4454	0.0366	0.3640	0.5242	0.7539	0.5471	0.8175
Sphinganine	0.4601	0.6348	0.8071	0.1967	0.4718	0.8566	0.5940
Sphingosine	0.1616	0.3544	0.1126	0.3627	0.6510	0.1630	0.6425
Uridine	0.0005	0.1098	0.6389	0.3183	0.0233	0.5800	0.2985
Hypoxanthine	0.5886	0.0102	0.1947	0.8182	0.2342	0.5157	0.6802
Uric acid	0.4730	0.1708	0.3359	0.5365	0.3956	0.2830	0.9972
Allantoin	0.8965	0.1600	0.3685	0.1535	0.3038	0.6694	0.7520

**Fig 3:**
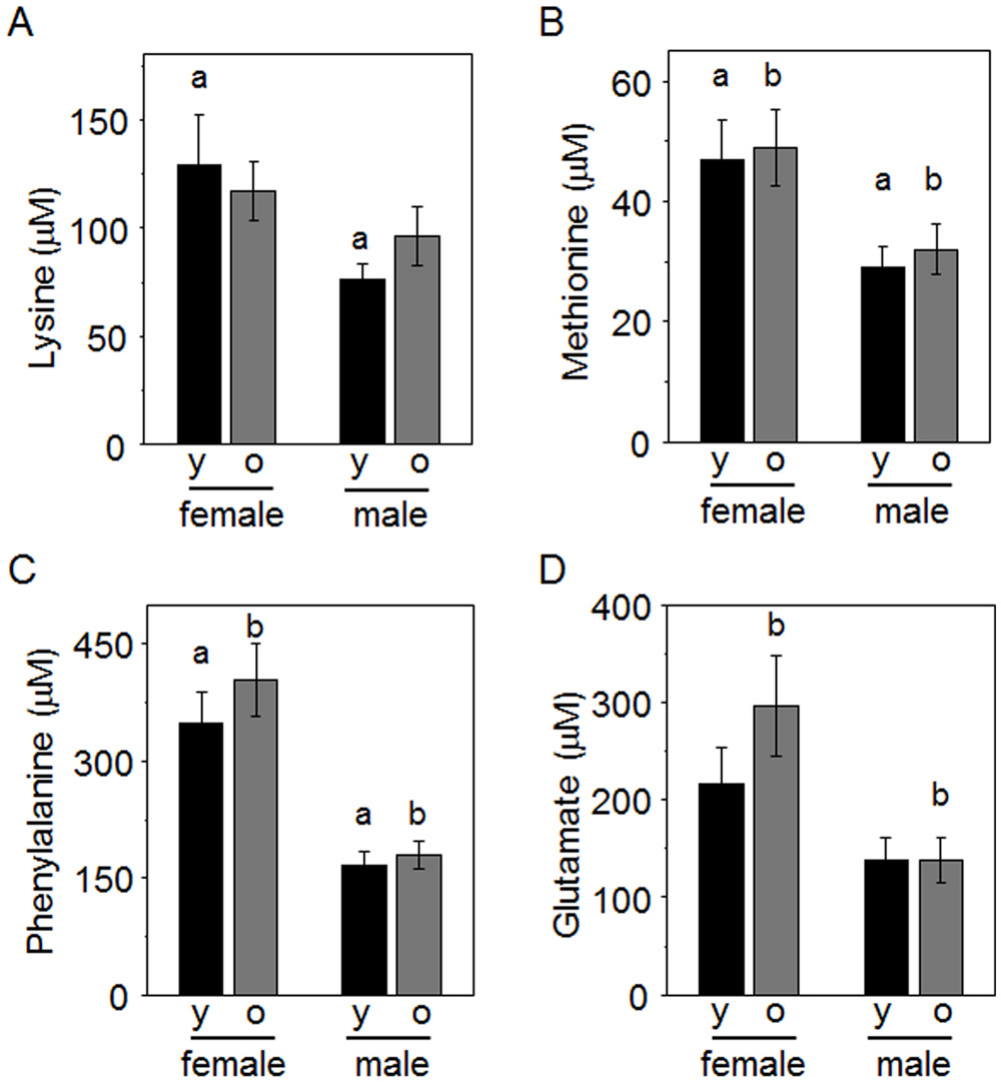
Metabolites higher in female than male marmosets. Female and male marmosets were grouped according to age as younger (y, 2 to 7 y) and older (o, 8 to 15 y). Following ANOVA, post-hoc tests showed differences between groups as indicated by a, female versus male in younger marmosets; b, female versus male in older marmosets. Group sizes were: females, 12 y, 12 o: males 13 y, 13 o.

**Fig 4:**
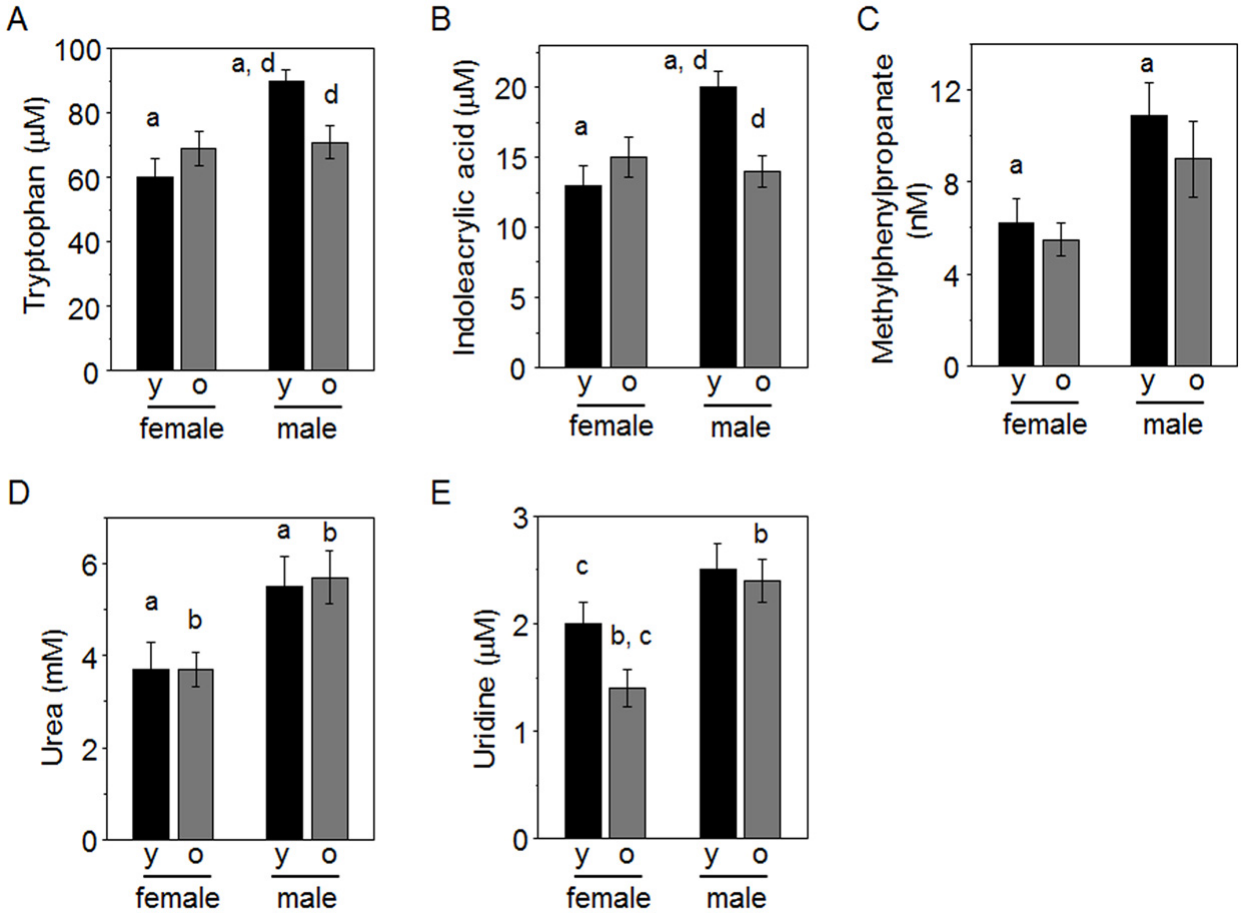
Metabolites higher in male than female marmosets. Female and male marmosets were grouped according to age as younger (y, 2 to 7 y) and older (o, 8 to 15 y). Following ANOVA, post-hoc tests showed differences between groups as indicated by a, female versus male in younger marmosets; b, female versus male in older marmosets; c, younger versus older females; d, younger versus older males. Group sizes were: females, 12 y, 12 o: males 13 y, 13 o.

A small number of metabolites were significantly different between males and females by ANOVA; Fig 3 shows metabolites higher in females and Fig 4 shows metabolites higher in males. These include some essential amino acids (Lys, Met and Phe in Fig 3; Trp in Fig 4), a non-essential amino acid (glutamate, Glu; Fig 3), amino acid metabolites (indoleacrylic acid, methylphenylpropanoate; Fig 4), a protein nitrogen degradation product (urea, Fig 4) and a pyrimidine metabolite, uridine. Other amino acids (His, citrulline) were not significantly higher in females but had p <0.1, indicating a trend. A vitamin essential for amino acid metabolism (pyridoxine) was not significantly higher in males, but also had p <0.1, indicating a trend. Sphingosine and sphinganine were not significantly different but had p <0.1, indicating a trend for sphingosine to be higher in males and sphinganine to be higher in females. No differences were apparent for the environmental chemicals that were measured. Analysis using ANCOVA showed that uridine was significantly different by sex after adjusting for age and body mass (Table 3). Other metabolites that were significant by ANOVA were also significant by ANCOVA, except lysine, which lost significance after adjusting for age and body mass.

**Fig 5:**
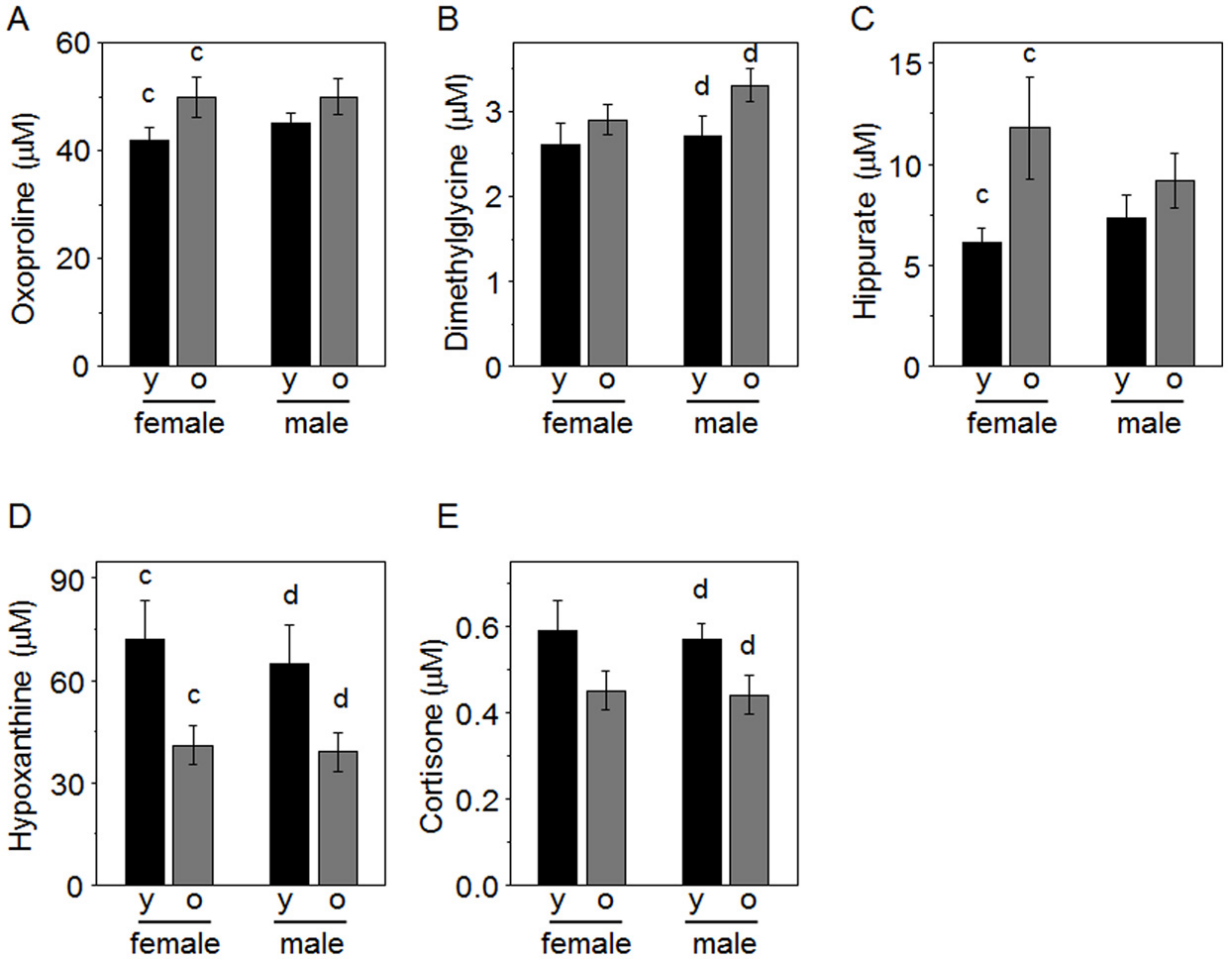
Metabolites differing by age in marmosets. Female and male marmosets were grouped according to age as younger (y, 2 to 7 y) and older (o, 8 to 15 y). A-C, metabolites higher in older than younger. D and E, metabolites higher in younger than older. Following ANOVA, post-hoc tests showed differences between groups as indicated: c, younger versus older females; d, younger versus older males. Group sizes were: females, 12 y, 12 o: males 13 y, 13 o.

With ANOVA, no differences were observed with age for amino acids or environmental chemicals. Three amino acid metabolites (oxoproline, dimethylglycine and hippurate) were higher in older marmosets in both females and males (Fig 5A–5C, Table 2). Two metabolites functioning in fatty acid metabolism by mitochondria, carnitine and acetylcarnitine, had p = 0.069 and p = 0.060, respectively, indicating trends to be higher in the older animals; especially, acetylcarnitine was significantly higher (p<0.05) in older females than younger females. Hypoxanthine, a purine metabolite, and cortisone, a stress hormone, were lower in older marmosets (Fig 5D and 5E, Table 2).

With adjustment for sex and body mass in ANCOVA, Trp and Gln were significantly associated with age (Table 3). In addition to the amino acid metabolites that were significant by ANOVA, 5-hydroxytryptophan, indoleacrylic acid, 3-indolepropionic acid and homogentisate, differed by age after adjustment for sex and body mass. ANCOVA also showed carnitine and acetylcarnitine, as well as hypoxanthine, were different by age after adjustment for sex and body mass (Table 3).

Sex differences for young marmosets were significant for urea, Lys, Met, Phe, Trp, indoleacrylic acid, methylphenylpropanoate, sphingosine and sphinganine (Table 2). Sex differences for older marmosets were significant for urea, Met, Phe, Glu, uridine and allantoin.

Age differences for females included oxoproline, hippurate, carnitine, uridine, hypoxanthine and allantoin. Of these, oxoproline, hippurate and carnitine were higher in the older females while uridine, hypoxanthine and allantoin were lower in the older females. Age differences for males included cortisone, Trp, indoleacrylic acid, dimethylglycine, sphingosine and hypoxanthine. Of these, only dimethylglycine was increased in the older males; Trp, indoleacrylic acid, sphingosine and hypoxanthine were decreased in older males.

Interactions between sex and age were seen for Trp, indoleacrylic acid and allantoin by ANOVA, but only Trp and indoleacrylic acid were significant by ANCOVA. ANCOVA also showed significant sex and body mass interactions for His, Trp, Glu, betaine and uridine, and significant age and body mass interactions for creatinine, indoleacrylic acid and indolelactate. Significant interaction of sex, age and body mass was observed for phenylacetic acid (Table 3).

### Metabolic Pathways Associated with Metabolites that Are Different by Species, Sex and Age

Amino acid differences were studied in detail for 6 primate species [37] and are converted to common units for comparison to marmosets in Table 4. Results show that average values for the aromatic amino acids Trp and Phe in marmoset plasma are higher than corresponding values for any of the other primate species, while arginine, asparagine and threonine values are lower than other primate species.

**Table 4:** Amino acid comparisons between common marmosets and other primates.

Amino Acid	Normalized Marmoset Data from Table 2 Mean±SD	Human Mean value (Table 2 value for normalization)	Squirrel Monkeys Mean±SE	Stump-tailed Macaques Mean±SE	Rhesus Monkey Mean±SE	Talapoin Monkeys Mean±SE	Chimpanzees Mean±SE
Arginine	42 ± 32	72 (80)	85 ± 15	86 ± 6	99 ± 11	85 ± 7	68 ± 5
Asparagine	12 ± 15	N/A (51)	N/A	45 ± 2	42 ± 2	111 ± 9	41 ± 3
Citrulline	31± 16	31 (28)	35 ± 4	42 ± 6	26 ± 5	22 ± 2	25 ± 2
Glutamate	55± 38	23 (35)	188 ± 28	101 ± 12	93 ± 5	139 ± 10	61 ± 3
Glutamine	642 ± 164	463 (535)	904 ± 100	466 ± 46	468 ± 3	384 ± 18	389 ± 16
Histidine	105 ± 31	52 (77)	110 ± 8	88 ± 12	85 ± 3	99 ± 8	63 ± 3
Leucine/Isoleucine	63 ± 31	156 (111)	113 ± 7	124 ± 14	114 ± 12	157 ± 18	60 ± 3
Lysine	152 ± 83	117 (171)	107 ± 13	186 ± 26	183 ± 16	194 ± 17	169 ± 10
Methionine	36 ± 18	24 (22)	60 ± 3	26 ± 3	18 ± 3	28 ± 1	20 ± 2
Phenylalanine	276 ± 154	47 (48)	69 ± 4	50 ± 4	52 ± 5	69 ± 2	46 ± 2
Proline	130 ± 48	195 (151)	176 ± 35	243 ± 38	179 ± 11	107 ± 9	218 ± 6
Taurine	158 ± 56	32 (48)	284 ± 70	205 ± 31	202 ± 12	287 ± 33	71 ± 6
Threonine	65 ± 19	103 (50)	178 ± 13	85 ± 8	82 ± 6	113 ± 10	97 ± 7
Tryptophan	95 ± 28	40 (53)	61 ± 7	36 ± 2	32 ± 3	40 ± 3	50 ± 3
Tyrosine	50 ± 23	44 (50)	72 ± 6	58 ± 4	40 ± 5	56 ± 3	52 ± 3

The original data of Peters et al, expressed as mg/dL, were converted to micromolar concentrations. To facilitate comparisons, marmoset data in this Table were normalized based upon the human concentration for the respective amino acid in Table 2 (given in parentheses) and the corresponding value provided by Peters et al. All concentrations are expressed as micromolar values.

**Fig 6:**
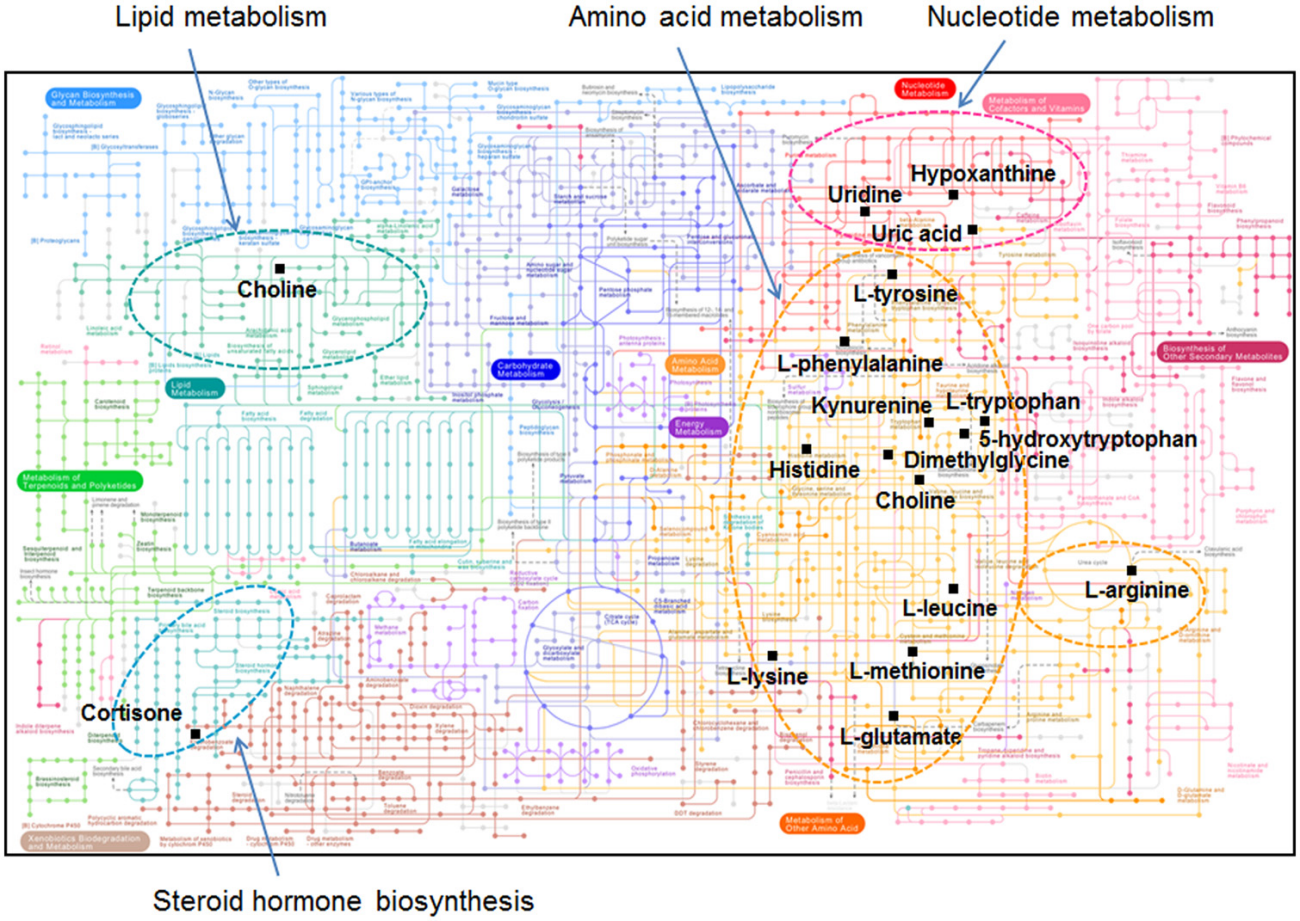
Visualization of metabolic pathways in marmosets that differed from ranges for human values in HMDB. Quantitatively different metabolites were visualized in the KEGG metabolic pathways. (http://www.genome.jp/kegg/pathway.html#metabolism). Black squares indicate quantitatively different metabolites matched in the metabolic pathways including lipid metabolism, amino acid metabolism, nucleotide metabolism and steroid hormone biosynthesis.

Metabolic pathways were visualized using KEGG pathway analysis for quantitatively different metabolites for age and sex. These include several amino acids and amino acid metabolites (Fig 6). Smaller numbers of other metabolites differed and included pathways for nucleotide metabolism, lipid metabolism, and steroid hormone biosynthesis (Fig 6).

## Discussion

Studies of conservation of genetic sequences has been very useful to understand evolution of species; in principle, conservation of metabolic pathways and intermediate concentrations could similarly provide useful information but has not been extensively developed. In our earlier study of seven mammalian species, non-targeted plasma metabolomics showed greater overlap of marmoset with rhesus macaques and humans than with rodentia and artiodactyla [18]. That comparison was qualitative, however, based upon the number of common metabolites detected. Although those metabolites detected matched metabolites in 137 out of 146 pathways in KEGG, more than half did not have matches to known chemicals in metabolomics databases. In that study, we used probability-based modularity clustering as a means to analyze chemicals according to the significance of their association with other chemicals [38]. By setting criteria to classify chemicals according to significance of within-species correlations and between-species correlations, a distribution was established in which modules of chemicals that have similar variation among species were discriminated from chemicals that have greater variation among species than within species. The results showed a large module enriched in endogenous metabolites, including leucine/isoleucine, citrulline, cystine, other amino acids and common intermediary metabolites. Another large module was enriched in environmental chemicals (e.g., pirimicarb and di-N-butyl phthalate) and metabolites involved in chemical and immunological defense (GSH, methionine and glutamine). Based upon this, we hypothesized that interspecies quantitative differences in central metabolites, metabolic degradation products and environmental chemicals, may be useful to improve understanding of speciation among primates. However, this is experimentally difficult to test because of the need to have large numbers of different species with good characterization of age, health and environmental parameters.

The present study was designed to gain an understanding of quantitative differences between marmosets and humans by measuring metabolites selected to include essential nutrients and other central metabolites as well as metabolic degradation products and environmental chemicals. The results provide no support for the interpretation derived from the probability-based modularity clustering that xenobiotics and non-essential metabolites will be more useful for studies of speciation; in fact the results suggest the opposite, that central metabolites are likely to be most useful for studies of speciation. About half of the plasma metabolite concentrations measured were significantly different when plasma samples were analyzed using the same analytical platform. Thus, the data indicate that enough quantitative differences exist to develop this approach as a complement to genetic studies of evolution.

Metabolites that differed between marmosets and humans included seven essential amino acids, one non-essential amino acid, five amino acid metabolites, two metabolites involved in 1-carbon metabolism, bilirubin, and uric acid. These metabolites were visualized in KEGG metabolic pathways (Fig 6) and mainly associated with amino acid metabolism and nucleotide metabolism. These are important in protein metabolism, nitrogen balance and cellular turnover, and systematic analysis could therefore be useful in study of these aspects of the comparative biology of primates. Additionally, differences in these pathways could be important in understanding susceptibility to metabolic, renal, hepatic or other diseases.

In a recent untargeted metabolomics study to study the effect of a semi-synthetic, purified diet on plasma metabolomics in marmosets [39], we found that methionine, leucine/isoleucine, lysine and threonine were higher on the purified diet while phenylalanine was lower. These results show that plasma metabolomics in marmosets is dependent upon diet so that prediction of concentrations in wild populations cannot readily be made. As found earlier [33], the present results show that Phe concentrations were higher in marmosets than in humans while results obtained here show that Tyr is lower in humans and the degradation product, homogentisate, is lower in marmosets. This could indicate that metabolism of Phe to Tyr is limited in the captive population, as would occur with tetrahydrobiopterin insufficiency [40]. Alternatively, genetic differences could occur between marmosets and humans in phenylalanine hydroxylase activity. In contrast, Trp levels were comparable to human values but three metabolites of Trp (kynurenine, 5-hydroxytryptophan and indolepropionic acid) were higher in marmosets. This result could be associated with ketamine used to sedate animals before blood collection as described in method. The previous human metabolomics study showed that ketamine altered mitochondrial fatty acid metabolism in patients with bipolar depression [41], and we also found that ketamine was strongly correlated with several metabolites associated with Trp pathways in our metabolome-wide association study (MWAS) (unpublished data). The effect of ketamine on marmoset metabolomics is currently under investigation. Trp metabolism is complex because toxic metabolites can be generated by the microbiome [42], and Trp serves as a precursor for seratonin [43], melatonin [44, 45] and niacin [46, 47], and also supports homeostasis of the immune system [48]. Because of the central roles of these metabolites as precursors in signaling and immune defenses, these differences could be important in diseases affecting marmosets. Additionally, Trp restriction has been linked to increased longevity in rodents [49], indicating that studies of Trp utilization in marmosets may be useful to understand associated mechanisms of aging.

Plasma Met was also higher in marmosets than in humans. Met restriction has been associated with increased longevity, linked to essential amino acid balance and the mTOR pathway [50, 51]. The Met product, homocysteine, has been linked to cardiovascular disease (CVD), but efforts to protect against CVD by altering this pathway have been unsuccessful [52]. Met is also an important precursor for S-adenosylmethionine synthesis and related methylation reactions involved in detoxification and epigenetic mechanisms [53, 54], as well as functioning as a precursor for Cys, GSH and hydrogen sulfide generation [55, 56]. Met concentrations were higher in females than males, and this was significant for younger and older marmosets. Related metabolites functioning as 1-carbon donors, choline and carnitine, were lower in marmosets than in humans but did not differ between females and males. Instead, carnitine and dimethylglycine were higher in older marmosets. Thus, the data show a complex age and sex dependency for Met and related 1-carbon metabolism, which will require more specific targeted study.

Arg was lower and His was higher in marmosets than in humans. Both are required for protein synthesis; Arg is also a precursor for nitric oxide, polyamines, urea and creatine, and His is a precursor for histamine. Additional studies will be needed to determine whether diet, genetics or both account for the differences between species. The above discussion highlights differences worthy of more detailed studies; overall, however, the data support the utility of marmosets for use as a model for experimental investigation of mechanisms of aging and human disease. The data show that all of the vitamin metabolites in marmosets overlapped with ranges found in humans, but riboflavin, pantothenate and biotin were significantly lower and nicotinamide, pyridoxine and pyridoxamine were higher. The data show that essential amino acid balances differ from humans, suggesting that amino acid metabolism may be a critical area for research on mechanisms of longevity in non-human primates. The data show that non-essential amino acid concentrations are very similar to human values; only taurine differed significantly. Measures of bilirubin and uric acid show that marmosets effectively eliminate these metabolites; significantly higher concentrations of uridine and hypoxanthine in marmosets suggest that related metabolic pathways or transport systems may differ, perhaps reflecting adaptation of marmosets to available habitat. Additionally, higher concentrations of carnitine and acetylcarnitine in older marmosets are consistent with extensive research implicating declining mitochondrial function in aging [57–59]. Of potential importance for carnitine, pathway analysis of marmosets on a purified diet showed effects on carnitine metabolism [39], indicating that changes in diet also impact related fatty acid metabolism.

How the results of this study on captive marmosets would compare to wild marmosets has not been evaluated. Animal husbandry to assure health of animals in captivity includes optimization of diet, avoidance of stressful environmental conditions, and medically treating or euthanizing sick animals. Because of this, complete agreement is not expected. On the other hand, wild and captive marmosets differ in body mass, and the ANCOVA results showed that body mass had little impact on which metabolites were significantly different according to sex or age (Table 3). These results suggest that the physiologic differences between sexes and between young and old marmosets may represent characteristics of the species and be useful for validation of studies on wild populations. Metabolomics studies of wild marmosets are needed to evaluate these differences.

In summary, quantification of 52 metabolites and 3 environmental chemicals in plasma of common marmosets indicates that differences in metabolite concentrations may provide a useful complement to non-targeted metabolomics for studies of diet and environment interactions in primate evolution. Such data could provide a new dimension to studies of primate evolution by allowing functional integration of the genome with environmental factors. Differences in essential amino acid concentrations indicate that more detailed studies are needed to evaluate underlying factors, which could contribute to food utilization as well as health and aging. The research highlights a need for studies of quantitative as well as non-targeted metabolomics to advance understanding of the natural biology of primates.

## Supporting Information

S1 Table Metabolomics data of 50 common marmosets. Plasma collected from marmoset was analyzed for metabolomics by high-resolution mass spectrometry as described in Methods. The mass spectrometry data includes ion mass (mass to charge, *m/z*), retention time (sec) and abundance (intensity) of 58 metabolites. The information of sex and age on 50 individuals is indicated on top.
(XLSX)

## Abbreviations

AA: Amino acids
HMDB: Human Metabolomics Database
LC-MS: liquid chromatography-mass spectrometry
*m/z*: mass-to-charge
rt: retention time
