# Correction: Metabolic Characterization of the Common Marmoset (*Callithrix jacchus*)

**DOI:** 10.1371/journal.pone.0147880

**Published:** 2016-01-22

**Authors:** Young-Mi Go, Yongliang Liang, Karan Uppal, Quinlyn A. Soltow, Daniel E. L. Promislow, Lynn M. Wachtman, Dean P. Jones

The creatinine values in Tables 1 and 2, and S1 Table are incorrect. Please see the corrected tables, with the correct creatinine values, here.

**Table 1:** Plasma metabolite concentrations in common marmosets.

Metabolite	*m/z*	rt	Marmosets Mean±SD	Humans Mean±SD	Human metabolomics Database
**Health indicators**					
Glucose (mM)	203.0512	57	4.0 ± 1.4	4.0 ± 0.9	3.9 to 6.1
Creatine (μM)	132.0759	52	38 ± 15	52 ± 35	8.4 to 65
Creatinine (μM)	114.0654	97	38.4 ± 15.1	102 ± 16*	56 to 109
Urea (mM)	121.0711	58	4.7 ± 2.1	3.2 ± 1.6*	4 to 9
Cortisol (μM)	363.2143	160	10.2 ± 4.1	0.49 ± 0.25*	0.028 to 0.66
Cortisone (μM)	361.1991	174	0.51 ± 0.19	0.010 ± 0.004*	0.022 to 0.075
Bilirubin (μM)	585.2668	350	1.9 ± 1.1	5.7 ± 6.4*	8 to 15
**Vitamins and Coenzymes**					
Riboflavin (nM)	377.1468	215	6.0 ± 5.7	15.5 ± 0.02*	5.4 to 28
Thiamine (μM)	265.1164	69	0.64 ± 0.62	0.39 ± 0.3	0.09 to 0.28
Niacin; nicotinic acid (μM)	124.0398	45	17.5 ± 7.0	29.8 ± 29.0	43 to 55
Nicotinamide (μM)	123.0544	326	0.43 ± 0.18	0.27 ± 0.15*	0.43 to 0.45
Methylnicotinic acid (nM)	138.0540	64	16 ± 9	20.0 ± 19.9	N/A
Pyridoxine (nM)	170.0714	61	59 ± 43	9.7 ± 6.1*	7 to 60
Pyridoxal (nM)	168.0643	93	230 ± 110	N/A	200 to 300
Pyridoxamine (nM)	169.0950	101	71 ± 56	0.14 ± 0.09*	126 to 202
Pantothenic acid (μM)	220.1761	231	4.1 ± 1.8	7.57 ± 7.22*	4.5 to 5.3
Biotin (nM)	245.0980	289	2.7 ± 1.1	4.5 ± 3.3*	0.6 to 1.9
**Amino Acids**					
Arginine (μM)	175.1178	59	40 ± 29	72 ± 33*	60 to 140
Histidine (μM)	178.0575	51	71 ± 21	52 ± 14*	75 to 143
Leucine/Isoleucine (μM)	176.0646	59	88 ± 43	156 ± 56*	155 to 355
Lysine (μM)	191.0755	48	104 ± 57	117 ± 41	178 to 434
Methionine (μM)	150.0568	79	39 ± 20	24 ± 6*	25 to 35
Phenylalanine (μM)	166.0851	57	271 ± 151	47 ± 15*	48 to 88
Threonine (μM)	120.0646	88	132 ± 39	103 ± 33*	102 to 260
Tryptophan (μM)	205.0958	56	72 ± 21	40 ± 11*	44 to 78
Asparagine (μM)	133.0679	92	18 ± 15	N/A	16 to 57
Citrulline (μM)	176.1018	63	34 ± 18	31 ± 14	27 to 38
Glutamate (μM)	148.0594	70	36 ± 25	23 ± 13	24 to 145
Glutamine (μM)	147.0758	56	553 ± 141	463 ± 317	490 to 645
Proline (μM)	116.0698	54	168 ± 62	195 ± 66	168 to 239
Taurine (μM)	148.0027	52	105 ± 37	32 ± 13*	42 to 162
Tyrosine (μM)	182.0799	58	44 ± 20	44 ± 11	54 to 143
**Amino Acid Metabolites**					
5-Hydroxytryptophan (μM)	221.0907	62	0.41 ± 0.32	0.030 ± 0.012*	0.015 to 0.021
Indoleacrylic acid (μM)	188.0693	64	0.02 ± 0.01	0.01 ± 0.00*	N/A
Indolelactate (μM)	206.0797	101	4.98 ± 1.67	0.72 ± 0.30*	0.5 to 5
3-Indolepropionic acid (μM)	190.0849	202	9.11 ± 5.33	0.04 ± 0.04*	0.29 to 1.09
Kynurenine (μM)	209.0914	73	3.68 ± 1.47	1.84 ± 0.72*	1.5 to 1.7
Phenylacetate (nM)	137.0586	216	2.7 ± 2.0	2.78 ± 2.15	N/A
Methylphenyllactate (nM)	181.0847	167	14 ± 7	48 ± 64*	N/A
Methylphenylpropanoate (nM)	165.0899	257	8.0 ± 5.0	6.5 ± 3.8	N/A
Homogentisic acid (μM)	169.0436	106	0.15 ± 0.09	0.02 ± 0.01*	0.014 to 0.071
Oxoproline (μM)	130.0490	53	47 ± 11	62 ± 13*	13 to 161
Hippurate (μM)	180.0644	63	8.6 ± 5.8	6.6 ± 6.3	0 to 5
2-Aminobutyrate (nM)	104.0698	54	6.0 ± 4.0	4.6 ± 1.4*	N/A
**Lipid-Related Metabolites**					
Choline (μM)	104.1062	51	1.5 ± 0.5	0.85 ± 0.23*	8.7 to 12.5
Betaine (μM)	118.0854	54	123 ± 64	31 ± 13*	20 to 144
Dimethylglycine (μM)	104.0697	424	2.91 ± 0.75	2.28 ± 0.97*	1.8 to 3.7
Carnitine (μM)	162.1114	79	6.7 ± 2.2	66 ± 24*	26 to 79
Acetylcarnitine (μM)	204.1216	66	0.54 ± 0.30	N/A	3.2 to 7.6
Sphinganine (nM)	302.3034	331	4.8 ± 2.6	6.6 ± 6.3	11
Sphingosine (μM)	300.2877	510	0.74 ± 0.33	1.21 ± 0.38*	0.05 to 0.51
**Nucleotide-Related Metabolites**					
Uridine (μM)	245.0757	95	2.11 ± 0.84	0.91 ± 0.68*	2.9 to 3.3
Hypoxanthine (μM)	137.0448	59	54 ± 34	3.9 ± 3.6*	1.3 to 54.5
Uric acid (μM)	169.0342	54	84 ± 73	214 ± 54*	238 to 506
Allantoin (μM)	159.0502	63	2.2 ± 1.1	N/A	1.0 to 3.2
**Environmental Chemicals**					
Triethylphosphate (nM)	183.0768	382	14.5 ± 3.4	10.8 ± 9.5	N/A
Pirimicarb (nM)	239.1475	548	0.7 ± 0.4	0.80 ± 0.36	N/A
Dibutylphthalate (nM)	279.1573	381	3.0 ± 1.0	14.0 ± 44.4	N/A

*Levels in common marmosets significantly different from the levels in human, *p* < 0.05, following Bonferroni correction.

Plasma metabolites of common marmosets (n = 50) quantified by LC-MS are included along with measures for human plasma (n = 80) obtained with the same method. Ranges of concentrations of metabolites in human plasma reported by HMDB are also included for comparison. (*m/z*, mass-to-charge, rt, retention time). N/A, data not available.

**Table 2:** Marmoset plasma metabolite concentrations according to age group and sex.

Metabolite	Females<8 y	Females ≥8 y	Males <8y	Males ≥8y
**Clinical measures**				
Glucose (mM)	4.8 ± 1.8^a^	4.1 ± 1.1	3.5 ± 1.3^a^	3.8 ± 1.4
Creatine (μM)	31 ± 13	43 ± 12	38 ± 11	42 ± 20
Creatinine (μM)	34.4 ± 15.5	35.1 ± 10.6	42.7 ± 6.8	81 ± 18
Urea (mM)	3.7 ± 2.0^a^	3.7 ± 1.3^b^	5.5 ± 2.4^a^	5.7 ± 2.1^b^
Cortisol (μM)	11 ± 4	9 ± 5	11 ± 3	10 ± 4
Cortisone (μM)	0.59 ± 0.24	0.45 ± 0.16	0.57 ± 0.13^d^	0.44 ± 0.16^d^
Bilirubin (μM)	2.0 ± 1.1	2.1 ± 1.0	1.5 ± 0.8	1.9 ± 1.3
**Vitamins and Coenzymes**				
Riboflavin (nM)	5 ± 5	11 ± 10	4 ± 4	6 ± 4
Thiamine (μM)	0.61 ± 0.37	0.95 ± 0.81	0.37 ± 0.27	0.57 ± 0.68
Niacin; nicotinic acid (μM)	17 ± 6	19 ± 6	19 ± 10	15 ± 4
Nicotinamide (μM)	0.42 ± 0.23	0.46 ± 0.17	0.41 ± 0.16	0.42 ± 0.18
Methylnicotinic acid (nM)	20 ± 10	14 ± 6	17 ± 11	14 ± 6
Pyridoxine (nM)	33 ± 14	57 ± 39	63 ± 37	76 ± 57
Pyridoxal (nM)	0.19 ± 0.09	0.25 ± 0.12	0.28 ± 0.11	0.20 ± 0.10
Pyridoxamine (nM)	87 ± 83	61 ± 25	57 ± 39	75 ± 57
Pantothenic acid (μM)	4.2 ± 0.9	3.5 ± 2.1	4.0 ± 1.4	4.5 ± 2.4
Biotin (nM)	3.1 ± 1.4	2.5 ± 1.1	2.9 ± 1.2	2.2 ± 0.7
**Amino Acids**				
Arginine (μM)	35 ± 28	43 ± 28	32 ± 21	48 ± 36
Histidine (μM)	74 ± 27	81 ± 21	59 ± 14	72 ± 19
Leucine/Isoleucine (μM)	99 ± 41	93 ± 58	87 ± 37	74 ± 33
Lysine (μM)	129 ± 82^a^	117 ± 48	76 ± 28^a^	96 ± 49
Methionine (μM)	47 ± 23^a^	49 ± 22^b^	29 ± 13^a^	32 ± 15^b^
Phenylalanine (μM)	348 ± 141^a^	404 ± 158^b^	167 ± 61^a^	180 ± 62^b^
Threonine (μM)	146 ± 58	135 ± 33	125 ± 31	124 ± 29
Tryptophan (μM)^e^	60 ± 20^a^	69 ± 19	90 ± 13^a^^,^^d^	71 ± 19^d^
Asparagine (μM)	16 ± 8	12 ± 11	23 ± 17	18 ± 18
Citrulline (μM)	35 ± 19	42 ± 17	32 ± 12	29 ± 20
Glutamate (μM)	216 ± 131	296 ± 180^b^	138 ± 83	138 ± 82^b^
Glutamine (μM)	509 ± 135	582 ± 168	519 ± 125	594 ± 130
Proline (μM)	169 ± 71	194 ± 58	158 ± 50	155 ± 67
Taurine (μM)	102 ± 43	107 ± 30	101 ± 35	110 ± 42
Tyrosine (μM)	45 ± 19	53 ± 20	41 ± 16	39 ± 22
**Amino Acid Metabolites**				
5-Hydroxytryptophan (μM)	0.35 ± 0.19	0.56 ± 0.52	0.31 ± 0.16	0.42 ± 0.27
Indoleacrylic acid (μM)^e^	13 ± 5^a^	15 ± 5	20 ± 4^a^^,^^d^	14 ± 4^d^
Indolelactate (μM)	4.9 ± 2.2	4.7 ± 1.0	5.3 ± 1.3	5.0 ± 2.0
3-Indolepropionic acid (μM)	9.5 ± 3.8	8.5 ± 6.8	9.8 ± 3.7	8.7 ± 6.6
Kynurenine (μM)	4.1 ± 1.9	3.4 ± 1.1	3.6 ± 1.4	3.6 ± 1.5
Phenylacetate (nM)	3.3 ± 1.9	2.8 ± 1.7	1.7 ± 1.5	2.8 ± 2.5
Methylphenyllactate (nM)	12 ± 5	15 ± 8	12 ± 6	17 ± 8
Methylphenylpropanoate (nM)	6.2 ± 3.8^a^	5.5 ± 2.4	10.9 ± 5.0^a^	9.0 ± 6.0
Homogentisic acid (μM)	139 ± 77	198 ± 119	113 ± 62	143 ± 100
Oxoproline (μM)	42 ± 8^c^	50 ± 13^c^	45 ± 7	50 ± 12
Hippurate (μM)	6.1 ± 2.6^c^	11.8 ± 8.7^c^	7.3 ± 4.2	9.2 ± 4.8
2-Aminobutyrate (nM)	5.3 ± 3.3	8.0 ± 5.4	6.7 ± 2.7	5.5 ± 2.4
**Lipid-Related Metabolites**				
Choline (μM)	1.4 ± 0.5	1.6 ± 0.5	1.3 ± 0.5	1.5 ± 0.5
Betaine (μM)	139 ± 76	141 ± 79	116 ± 48	99 ± 45
Dimethylglycine (μM)	2.6 ± 0.9	2.9 ± 0.6	2.7 ± 0.7^d^	3.3 ± 0.7^d^
Carnitine (μM)	5.4 ± 1.4^c^	7.9 ± 2.6^c^	6.2 ± 1.9	7.0 ± 2.3
Acetylcarnitine (μM)	0.45 ± 0.15	0.57 ± 0.26	0.45 ± 0.22	0.65 ± 0.44
Sphinganine (nM)	6.0 ± 2.1^a^	5.0 ± 2.5	3.5 ± 2.6^a^	4.9 ± 2.7
Sphingosine (μM)	0.69 ± 0.34^a^	0.62 ± 0.33	1.00 ± 0.27^a^^,^^d^	0.69 ± 0.29^d^
**Nucleotide-Related Metabolites**				
Uridine (μM)	2.0 ± 0.7^c^	1.4 ± 0.6^b^^,^^c^	2.5 ± 0.9	2.4 ± 0.7^b^
Hypoxanthine (μM)	72 ± 40^c^	41 ± 20^c^	65 ± 40^d^	39 ± 21^d^
Uric acid (μM)	110 ± 120	60 ± 38	78 ± 41	78 ± 50
Allantoin (μM)^e^	2.7 ± 0.9^c^	1.5 ± 0.8^b^^,^^c^	2.0 ± 1.1	2.4 ± 1.4^b^
**Environmental Chemicals**				
Triethylphosphate (nM)	13.7 ± 3.7	14.8 ± 4.3	14.3 ± 2.7	15.0 ± 3.3
Pirimicarb (nM)	0.86 ± 0.49	0.63 ± 0.32	0.54 ± 0.33	0.73 ± 0.35
Dibutylphthalate (nM)	0.26 ± 0.14	0.25 ± 0.10	0.30 ± 0.13	0.33 ± 0.18

^a^Significant between younger females and younger males

^b^Significant between older females and older males

^c^Significant between younger females and older females

^d^Significant between younger males and older males

^e^Significant sex by age interaction

## Supporting Information

S1 Table Metabolomics data of 50 common marmosets. Plasma collected from marmoset was analyzed for metabolomics by high-resolution mass spectrometry as described in Methods. The mass spectrometry data includes ion mass (mass to charge, *m/z*), retention time (sec) and abundance (intensity) of 58 metabolites. The information of sex and age on 50 individuals is indicated on top.
(XLSX)
